# Chronic Endurance Exercise Impairs Cardiac Structure and Function in Middle-Aged Mice with Impaired Nrf2 Signaling

**DOI:** 10.3389/fphys.2017.00268

**Published:** 2017-05-03

**Authors:** Gobinath Shanmugam, Madhusudhanan Narasimhan, Robbie L. Conley, Thiagarajan Sairam, Ashutosh Kumar, Ronald P. Mason, Ramalingam Sankaran, John R. Hoidal, Namakkal S. Rajasekaran

**Affiliations:** ^1^Cardiac Aging and Redox Signaling Laboratory, Division of Molecular and Cellular Pathology, Department of Pathology, University of Alabama at BirminghamBirmingham, AL, USA; ^2^Department of Pharmacology and Neuroscience, Texas Tech University Health Sciences CenterLubbock, TX, USA; ^3^PSG Center for Molecular Medicine and Therapeutics, PSG Institute of Medical Sciences and Research, PSG HospitalsCoimbatore, India; ^4^Immunity, Inflammation, and Disease Laboratory, NIEHS/NIHRaleigh, NC, USA; ^5^Division of Pulmonary, Department of Medicine, University of Utah School of MedicineSalt Lake City, UT, USA; ^6^Division of Cardiovascular Medicine, Department of Medicine, University of Utah School of MedicineSalt Lake City, UT, USA; ^7^Center for Free Radical Biology, University of Alabama at BirminghamBirmingham, AL, USA

**Keywords:** Nrf2, diastolic dysfunction, cardiac hypertrophy, aging, endurance exercise

## Abstract

Nuclear factor erythroid 2 related factor 2 (Nrf2) signaling maintains the redox homeostasis and its activation is shown to suppress cardiac maladaptation. Earlier we reported that acute endurance exercise (2 days) evoked antioxidant cytoprotection in young WT animals but not in aged WT animals. However, the effect of repeated endurance exercise during biologic aging (WT) characterized by an inherent deterioration in Nrf2 signaling and pathological aging (pronounced oxidative susceptibility—Nrf2 absence) in the myocardium remains elusive. Thus, the purpose of our study was to determine the effect of chronic endurance exercise-induced cardiac adaptation in aged mice with and without Nrf2. Age-matched WT and Nrf2-null mice (Nrf2^−/−^) (>22 months) were subjected to 6 weeks chronic endurance exercise (25 meter/min, 12% grade). The myocardial redox status was assessed by expression of antioxidant defense genes and proteins along with immunochemical detection of DMPO-radical adduct, GSH-NEM, and total ubiquitination. Cardiac functions were assessed by echocardiography and electrocardiogram. At sedentary state, loss of Nrf2 resulted in significant downregulation of antioxidant gene expression (*Nqo1, Ho1, Gclm, Cat*, and *Gst-*α) with decreased GSH-NEM immuno-fluorescence signals. While Nrf2^−/−^ mice subjected to CEE showed an either similar or more pronounced reduction in the transcript levels of *Gclc, Nqo1, Gsr*, and *Gst-*α in relation to WT littermates. In addition, the hearts of Nrf2^−/−^ on CEE showed a substantial reduction in specific antioxidant proteins, G6PD and CAT along with decreased GSH, a pronounced increase in DMPO-adduct and the total ubiquitination levels. Further, CEE resulted in a significant upregulation of hypertrophy genes (*Anf, Bnf*, and β*-Mhc*) (*p* < 0.05) in the Nrf2^−/−^ hearts in relation to WT mice. Moreover, the aged Nrf2^−/−^ mice exhibited a higher degree of cardiac remodeling in association with a significant decrease in fractional shortening, pronounced ST segment, and J wave elevation upon CEE compared to age-matched WT littermates. In conclusion, our findings indicate that while the aged WT and Nrf2 knockout animals both exhibit hypertrophy after CEE, the older Nrf2 knockouts showed ventricular remodeling coupled with profound cardiac functional abnormalities and diastolic dysfunction.

## Introduction

Heart failure is one of the major health problems in the globe and is the leading cause of death in the United States, accounting for 1 in every 4 deaths. Among the elderly, there were approximately 610,000 deaths due to heart disease in 2015 in the U.S. (Mozaffarian et al., 2015). By 2025, the percentage of aged persons diagnosed with heart disease and associated mortality rate is projected to increase (Finegold et al., 2013). Age-associated oxidative stress (OS) is considered to be one of the principal risk factors for cardiovascular disease (CVD) due to its ability to induce changes including but not limited to signaling, inflammation, structural, and functional processes in cardiomyocytes (Bayes et al., 2006; Cui et al., 2012). These alterations when occurring for a chronic period under aging blunts both the adaptive as well as the regenerative ability of the heart leading to pathological remodeling and heart failure (Munzel et al., 2015; Rungatscher et al., 2015; Jeong et al., 2016; Kumar et al., 2016). Although pathogenic consequences of OS on ventricular dysfunction in failing hearts have received clinical attention (Takimoto and Kass, 2007; Fraccarollo et al., 2015), the mechanisms associated with antioxidant transcriptional signaling in response to a physical activity in an aging heart remains largely unknown.

There is compelling evidence demonstrating that ventricular remodeling, following acute stress is a beneficial adaptation. However, it turns as maladaptation under chronic stress conditions and leads to progressive heart failure in humans (Mann and Bristow, 2005). Aerobic exercise builds a physiologic form of hypertrophy in the myocardium with unaltered or improved systolic or diastolic function (Rawlins et al., 2009; Fernandes et al., 2015) and transient upregulation of remodeling biomarkers in experimental models (Konhilas et al., 2004; Fernandes et al., 2011). A recent finding revealed that high-endurance competitive events such as marathon, ultramarathon, triathlon, and very long distance bicycle races can result in momentary volume overload of the ventricle, with a subsequent increase in ejection fraction (EF) and augmented signaling of cardiac remodeling (Patil et al., 2012). Thus, it is widely considered that an appropriately timed physical exercise combined with the right amount and type can negate the pathological remodeling in response to myocardial infarction, and development of heart failure.

Earlier we reported that acute endurance exercise (2 days) evoked antioxidant cytoprotection in young WT animals whereas Nrf2^−/−^ young mice and aged WT mice that is intrinsically defective in Nrf2 signaling did not receive similar benefits as that of young WT (Gounder et al., 2012; Muthusamy et al., 2012). Moreover, we have demonstrated that moderate exercise regimens are found to be cardio-protective via priming the Nrf2-dependent antioxidant signaling and maintaining myocardial defense in WT animals (Gounder et al., 2012). Notably, longitudinal human and several animal studies have shown that age-associated cardiac function fall into two categories such as pathological (accumulated and/or forced changes over time) and normal (gradual deterioration) (Lakatta and Levy, 2003a,b; Dai and Rabinovitch, 2009; Pandey et al., 2017). Thus, it is increasingly important to understand the cardiac adaptations that accompany aging, both under normal and frailty conditions. Importantly, there is a lack of report on the effect of repeated endurance exercise during normal biological aging where there is an inherent deterioration in Nrf2 signaling and non-normative pathological aging (increased oxidative vulnerability due to Nrf2 absence). Thus, the purpose of this study was to determine the effects of chronic endurance exercise training-induced cardiac plasticity and how it is modulated in the presence and absence of Nrf2 in aging mice. Addressing this question is vital for a comprehensive understanding of CEE-regulated cardiac physiology and function and shed light on the magnitude of performance required to maintain cardiac homeostasis during both normal and abnormal myocardial aging. Here we demonstrated that chronic endurance exercise (CEE)-induced significant hypertrophic changes accompanied by ventricular dysfunction (diastolic) in WT mice. The magnitude of these changes was more pronounced in the age-matched Nrf2^−/−^ mice suggesting that Nrf2 limits the overt cardiac malformation and may preserve the cardiac function in a model of chronic EE. Collectively, these data expand our understanding of the cause and effect relationship between Nrf2-dependent redox mechanisms and CEE-induced cardiac pathology on aging.

## Materials and methods

### Reagents

DMPO was purchased from DOJINDO Molecular Technologies; Inc. Rockville, MD, USA. RNeasy kit, reverse transcription kit, and QuantiTect SYBR Green PCR kit were purchased from Qiagen Inc., Valencia, CA. The rabbit anti-Ubiquitin (ab7780), was procured from Abcam, Cambridge, MA, USA and mouse anti-GSH-NEM ab from EMD Millipore, USA (MAB3194) Secondary antibodies for immunofluorescence conjugated with Alexa fluor 488 anti-rabbit and anti-mouse were obtained from Life Technologies Corporation, NY, USA. Primers for qPCR were purchased from Integrated DNA Technologies, Coralville, IA. All other chemicals were purchased from Sigma-Aldrich unless otherwise stated.

### Animals

C57/Bl6J wildtype (WT) and Nrf2 knock out (Nrf2^−/−^) mice (>22 months of age, Male/Female) were used. WT Mice were purchased from the Jackson Laboratory (Maine, USA) and Nrf2^−/−^ mice were obtained from Dr. Li Wang (University of Utah, Salt Lake City USA). Both WT and Nrf2-KO mice were the litter mates obtained from heterozygous (Nrf2^+/−^ × Nrf2^+/−^) male and female parents with C57/Bl6 background. Mice were housed in the animal research facility at the University of Alabama at Birmingham. All cages housed not more than five mice each had free access to food (standard rodent diet) with water *ad libitum* were maintained under controlled conditions. Animal treatments and protocols were approved by the Institutional Animal Care and Use Committee (IACUC) of the University of Alabama at Birmingham.

### Chronic endurance exercise

WT and Nrf2^−/−^ mice (*n* = 3–6/group/experiment) were subjected to CEE on a treadmill. Briefly, mice were subjected to running on a treadmill for 6 weeks at 25 m/min; 12% grade, for 60 min per day. An equal number of mice were assigned to the sedentary group. After 6 weeks of CEE, mice were sacrificed by CO_2_ inhalation and the heart tissues were stored at −80°C for subsequent molecular analysis. Tissues were stored in RNA*later* (Sigma) at −80°C until further processing for real time PCR analysis.

### RNA isolation, reverse transcription and qRT-PCR

The RNA was isolated from the hearts of WT and Nrf2^−/−^ mice (CEE and sedentary) that were stored in RNA*later* using RNA extraction kits (Qiagen #74106). The RNA was quantified by NanoDrop 2000C Spectrophotometer and 1.25 μg was used to synthesize cDNA (Qiagen Reverse Transcription Kit Cat #205311) according to the manufacturer's instructions. Quantitative PCR was performed using 25–50 ng cDNA template in a final reaction mixture of 10 μL of SYBR green master mix (Qiagen #204054) with respective primer sets (1 pmol) (Table 1) and was amplified in a Roche light cycler 480. mRNA expression of the targets was quantified using Ct values, and expression fold change was calculated by normalization to the Ct of housekeeping gene *Gapdh* using the 2^−ΔΔCt^ method (Livak and Schmittgen, 2001).

**Table 1:** **List of primers used for qRT-PCR experiments**.

**Genes name**	**Sequences (5′–3′)**
*Anf F*	GCTTCCAGGCCATATTGGAG
*Anf R*	GGGGGCATGACCTCATCTT
*Arbp1F*	TGAGATTCGGGATATGCTGTTGG
*Arbp1R*	CGGGTCCTAGACCAGTGTTCT
*αMhc F*	GAGTGGGAGTTTATCGACTTCG
*aMhc R*	CCTTGACATTGCGAGGCTTC
*Bnf F*	GAGGTCACTCCTATCCTCTGG
*Bnf R*	GCCATTTCCTCCGACTTTTCTC
*α-Mhc F*	ACTGTCAACACTAAGAGGGTCA
*αMhc R*	TTGGATGATTTGATCTTCCAGGG
*Cat F*	GGAGGCGGGAACCCAATAG
*Cat R*	GTGTGCCATCTCGTCAGTGAA
*Gapdh F*	TGACCTCAACTACATGGTCTACA
*Gapdh R*	CTTCCCATTCTCGGCCTTG
*Gclc F*	GGACAAACCCCAACCATCC
*Gclc R*	GTTGAACTCAGACATCGTTCCT
*Gclm F*	CTTCGCCTCCGATTGAAGATG
*Gclm R*	AAAGGCAGTCAAATCTGGTGG
*Gpx1 F*	CCACCGTGTATGCCTTCTCC
*Gpx1 R*	AGAGAGACGCGACATTCTCAAT
*Gsr F*	CACGGCTATGCAACATTCGC
*Gsr R*	GTGTGGAGCGGTAAACTTTTTC
*Gstm4 F*	CTGAAGGTGGAATACTTGGAGC
*Gstm4 R*	GCCCAGGAACTGTGAGAAGA
*Gsta4 F*	TGATTGCCGTGGCTCCATTTA
*Gsta4 R*	CAACGAGAAAAGCCTCTCCGT
*G6pd F*	TCAGACAGGCTTTAACCGCAT
*G6pd R*	CCATTCCAGATAGGGCCAAAGA
*Ho-1 F*	AAGCCGAGAATGCTGAGTTCA
*Ho-1 R*	GCCGTGTAGATATGGTACAAGGA
*Nqo1 F*	AGGATGGGAGGTACTCGAATC
*Nqo1R*	TGCTAGAGATGACTCGGAAGG
*Pln F*	AAAGTGCAATACCTCACTCGC
*Pln R*	GGCATTTCAATAGTGGAGGCTC
*Serca2a F*	AAATGGGCAAAGTGTATCGACA
*Serca2a R*	CTTGATGACGGAGACAGATTCAC
*Sod-1 F*	AACCAGTTGTGTTGTCAGGAC
*Sod-1 R*	CCACCATGTTTCTTAGAGTGAGG
*Sod2 F*	TGGACAAACCTGAGCCCTAAG
*Sod2 R*	CCCAAAGTCACGCTTGATAGC

### Protein isolation and immunoblotting

WT and Nrf2^−/−^ mice (SED and CEE) heart tissues (40–50 mg) were homogenized using cytosolic extraction buffer (10 mM HEPES, 10 mM KCl, 0.1 mM EDTA, 0.5 mM MgCl_2_, with freshly prepared 0.1 mM phenyl methylsulfonyl fluoride (PMSF), 1 mM dithiothreitol and 1% Triton X-100, pH 7.9) and centrifuged at 5,000 rpm for 5–6 min. Protein concentrations were determined with the Bradford reagent (Bio-Rad, Hercules, CA, USA), and 10–15 μg of protein was resolved in 10–12% SDS-PAGE and transferred to polyvinylidene difluoride (PVDF) membranes (EMD Millipore Corp., Billerica, MA, USA). The membranes were blocked in Tris buffered saline-Tween 20 (TBST) containing 5–10% non-fat dry milk for 1 h and then incubated for overnight at 4°C with any one of these primary antibodies (Catalase #219010, Calbiochem, Germany), GAPDH (5174S, Cell Signaling Technology, Danvers, MA), glutathione peroxidase (GPX1; ab22604, Abcam Inc, USA), G6PD (NB100-236, Novus Biological), SOD1 (ab13498, Abcam Inc, USA) diluted with 2% bovine serum albumin in TBST. Horse radish peroxidase IgG conjugated secondary antibodies (anti-rabbit, Vector Laboratories, Burlingame, CA, USA) was incubated for 1 h followed by ECL–chemiluminescence (Pierce, Rockford, IL, USA) based detection was performed and images were obtained with an Amersham Imager 600 (GE Healthcare Life Sciences, Chicago, IL, USA). The densitometry signals were quantified by using ImageJ software and normalized to the intensity of GAPDH.

### Immunofluorescence staining

Myocardial samples prepared in optimal cutting temperature (OCT) medium were processed for immunofluorescence (IF). From each heart, 10 μm cryostat sections were placed on slides and incubated with 4% paraformaldehyde for 15 min and washed thrice with PBS. Sections were then permeabilized using 0.25% Triton X-100 followed by three washes with PBS. For GSH staining, after permeabilization, the sections were incubated in ethanol containing 10 mM N-Ethylmaleimide for 30 min and washed three times with PBS. This was followed by blocking in 5% normal goat serum in PBS for 1 h and appropriate primary antibodies incubations overnight at 4°C at the following concentrations: mouse anti GSH-NEM (1:500) and rabbit anti-Ubiquitin (1:500). The sections were then incubated with secondary antibody Alexa fluor 488 goat anti-rabbit/ mouse IgG (H+L) (1:1,000) followed by mounting with fluoroshield/DAPI (ab104139, Abcam, Cambridge, MA, USA). Images were captured using Nikon A1Confocal microscope (Nikon Instruments Inc.) at 60X magnification. Fluorescence signals from three different random areas per section were captured, and the intensity was quantified using Image J software (NIH) and expressed as relative fold change with respect to the control group.

### Anti-DMPO staining protocol

Following the final exercise session, WT and Nrf2^−/−^ (SED and CEE) mice were injected with DMPO dissolved in pyrogen-free saline i.p (1.5 g/kg total) in two doses at 1 h interval and then sacrificed 1 h after the second dose. Heart tissues were perfused with phosphate-buffered saline (PBS; pH 7.4) and fixed with 10% buffered zinc formalin for 24 h followed by incubation with 30% sucrose for 24 h and cryopreserved in liquid nitrogen. Frozen tissues were sectioned (10 μm) and blocked with 1% casein-PBS followed by incubation with mouse anti-DMPO primary antibody (5 μg/ml; kindly provided by Dr. Ronald P. Mason, NIEHS, NC). Sections were then washed with PBS and incubated with secondary antibody Alexa fluor 488 goat anti-rabbit IgG (H+L) (1:1,000; A11008, Life Technologies Corporation, NY, USA) for 1 h in the dark prior to mounting with DAPI (Mason, 2016). A Nikon A1Confocal microscope was used to capture the fluorescence signals and three random areas were imaged from each section at 60x magnification. Mean fluorescence intensity was obtained using Image J software (NIH) and was expressed as relative fold change with respect to the control group.

### Electrocardiogram measurements

Mice were anesthetized using 1.5–2% isoflurane mixed with 1 L/min O_2_ via facemask. The 3-lead isolated ECG electrodes attached to the limbs of each mouse were connected to ix-228/S data acquisition unit (iWorx systems, Inc.) with LabScribe 2 software. The signals were filtered between 3 and 500 Hz and the input range is within 5 mV. The signals were also digitized with 16-bit precision at a sampling rate of 1,000 samples/second. ECG signal was continuously recorded for 5 min analysis. Only data from recordings of stable ECG signals were used in the analyses. The data were then analyzed with Labchart 8 software (AD instruments). Waterfall Plots, average plots, PR, RR, QRS interval (s), S-T elevation (s), and R-Amplitude (v), Heart rate values were determined using LabChart Pro software (AD Instruments Inc. CO, USA). Four consecutive beats of ECG waveforms were averaged for analyses. When recording is completed, the anesthetic is turned off, the mouse is allowed to wake up and returned to its cage.

### Echocardiography imaging and analysis

A VisualSonics Vevo–2100 echocardiographic system with MS550 probe (30 μm resolution) was used to analyze cardiac function in WT and Nrf2^−/−^ mice under sedentary and CEE conditions. Isoflurane (1–2%) was used to maintain the mice in anesthetic condition for image acquisition. Hair in the chest was removed off using Nair (hair removal cream) and all images were acquired at 37°C with an average heart rate of 400–460 bpm. B-mode was used to obtain 2-dimensional real time long axis view images for strain analysis to measure EF and cardiac output. Short axis view images were captured using M-mode at the mid ventricular level and visualized by the presence of papillary muscles to measure ventricular dimension, fractional shortening, and wall thickness. Pulse wave color Doppler mode was used in apical four chamber view to measure the diastolic function of the heart. After the acquisition, all mice were recovered from the anesthesia procedure and returned to its cages.

### Statistical analysis

Mean values for relative gene expression or cardiac structure and functional parameters were expressed as mean ± *SD* for *n* = 4 in each experimental group. Statistical analysis for the comparison between the treatment and control groups was performed by one-way ANOVA followed by Tukey multiple comparison tests using GraphPad Prism software. Statistical significance was indicated where *p*-value was < 0.05.

## Results

### Chronic endurance exercise impacts the age-associated antioxidant gene and protein expression in Nrf2^−/−^ myocardium

**Figure 1:**
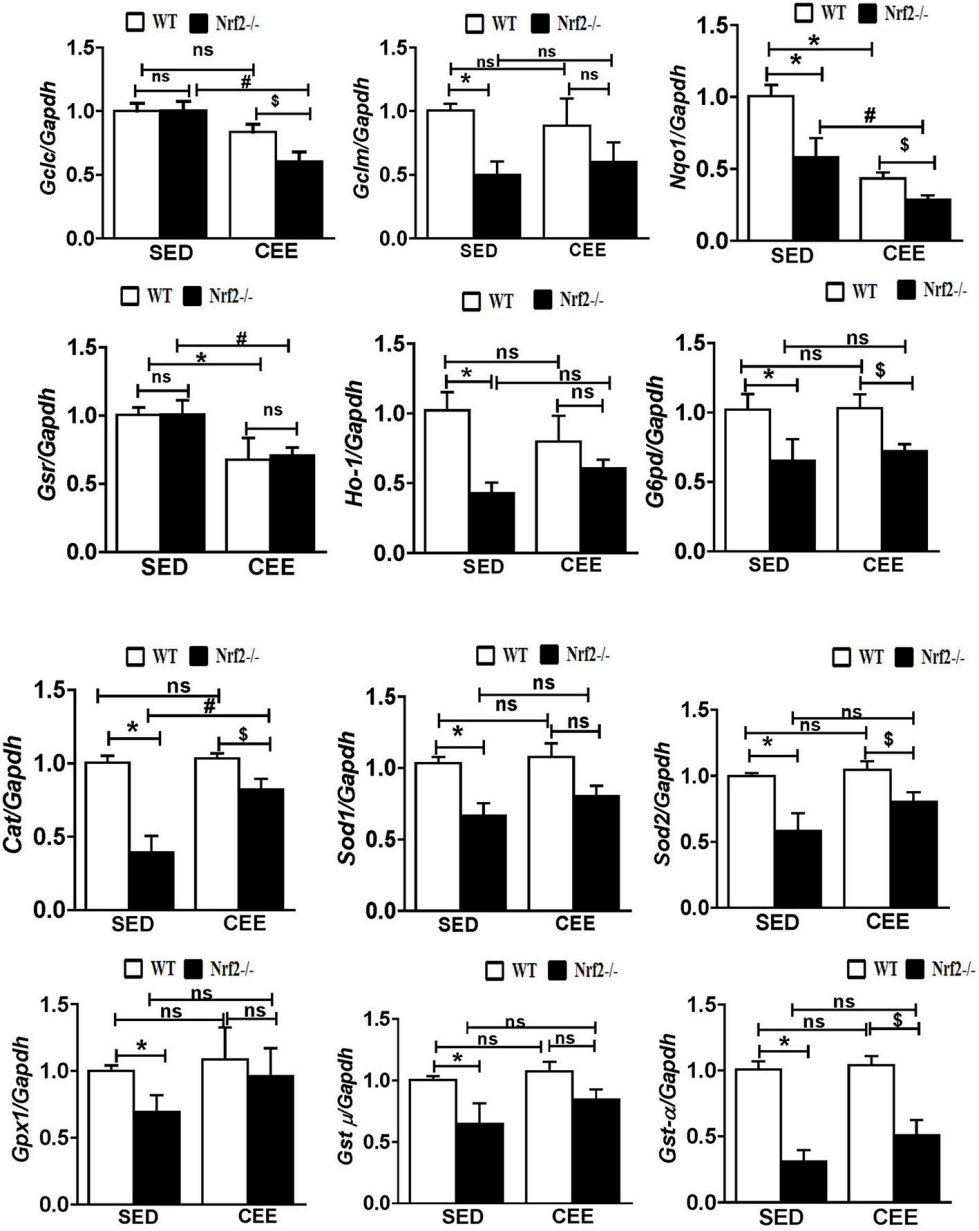
**Disruption of the antioxidant system upon chronic endurance exercise in aged Nrf2^**−/−**^ mice hearts**. mRNA expression of *Gclc, Gclm, Nqo1, Gsr, Ho-1, G6pd, Cat, Sod1, Sod2, Gpx1, Gst-*μ*, and Gst-*α were determined in WT and Nrf2^−/−^mice hearts by RT-qPCR. Gene expression was calculated by normalizing the Ct values with the housekeeping gene *Gapdh/Arbp1. N* = 4–6/ group. Results are expressed as mean ± *SD* and different symbols above the bars indicate statistically different mean values with the corresponding groups as assessed by one way ANOVA followed by Tukey multiple comparison tests (^*^P < 0.05 vs. WT-SED, ^#^*P* < 0.05 vs. Nrf2^−/−^-SED, ^*$*^*P* < 0.05 vs. WT CEE).

**Figure 2:**
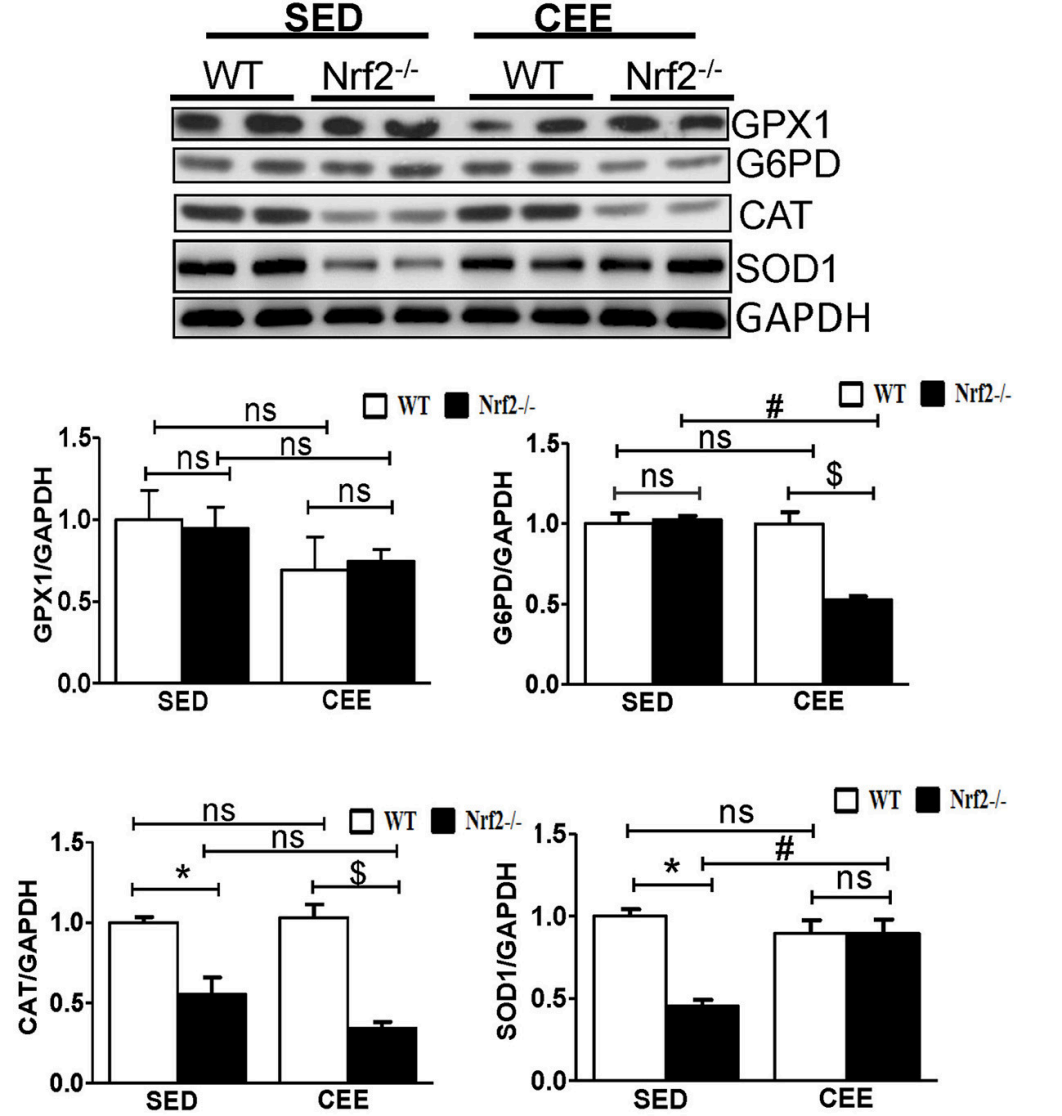
**Dysregulation of Antioxidant proteins in response to chronic endurance exercise in aged Nrf2^**−/−**^ mice hearts**. Antioxidant protein expression (GPX1, CAT, G6PD, and SOD1) were determined using immunoblotting with specific antibodies. The relative intensity signals were quantified using ImageJ software and normalized to GAPDH intensity and represented as histogram. Experiments were analyzed using one way ANOVA followed by Tukey multiple comparison tests and differences between the means were considered statistically significant if *P* < 0.05 (^*^ vs. WT-SED, ^#^ vs. Nrf2^−/−^-SED, ^*$*^ vs. WT CEE).

Numerous studies have reported that Nrf2 is a master transcriptional factor of antioxidant genes, regulating antioxidant gene expression through their antioxidant response element ARE binding sites (Gounder et al., 2012; Muthusamy et al., 2012; Pall and Levine, 2015). We and others have reported that aging decreases Nrf2 (Safdar et al., 2010; Gounder et al., 2012; Done et al., 2016) and thus to determine whether chronic endurance training exacerbates the age-associated alterations in antioxidant gene expression in the absence of Nrf2, we performed real time PCR analysis for the common Nrf2 target antioxidant gene expression in the aged WT and Nrf2^−/−^ mouse hearts. Under sedentary conditions, Nrf2 deficiency resulted in a significant decrease in the expression of *Nqo1, Gclm, Ho-1, Sod1, Gst-*α in Nrf2^−/−^ mice in relation to aged WT mice. At 22–24 months of age, homozygous Nrf2 knockout mice that had undergone 6 weeks of endurance exercise (*P* < 0.05) exhibited significantly greater downregulation in the expression of *Nqo1, Gclc, Gsr, and Gst-*α vs the age-matched WT littermates (Figure 1). The expression of other antioxidant genes such as *G6pd, Gpx1, Gst-*μ, *Sod1, Sod2, Cat* was unaltered in both WT and Nrf2^−/−^ mice in the CEE condition. Further, immunoblotting analyses revealed that the expression of cytoprotective antioxidant proteins, namely GPX1 and SOD1 remained unaltered in the hearts of Nrf2^−/−^ mice subjected to CEE compared to its WT counterparts. However, the expression of G6PD and CAT was significantly downregulated upon CEE in the hearts of Nrf2^−/−^ mice in relation to its WT counterparts (Figure 2). These results indicate that the age-associated alteration of antioxidant cascade downstream of Nrf2 pathway following endurance exercise is differential and selective.

### Prolonged exercise decreases GSH levels in aged Nrf2 deficient mice

**Figure 3:**
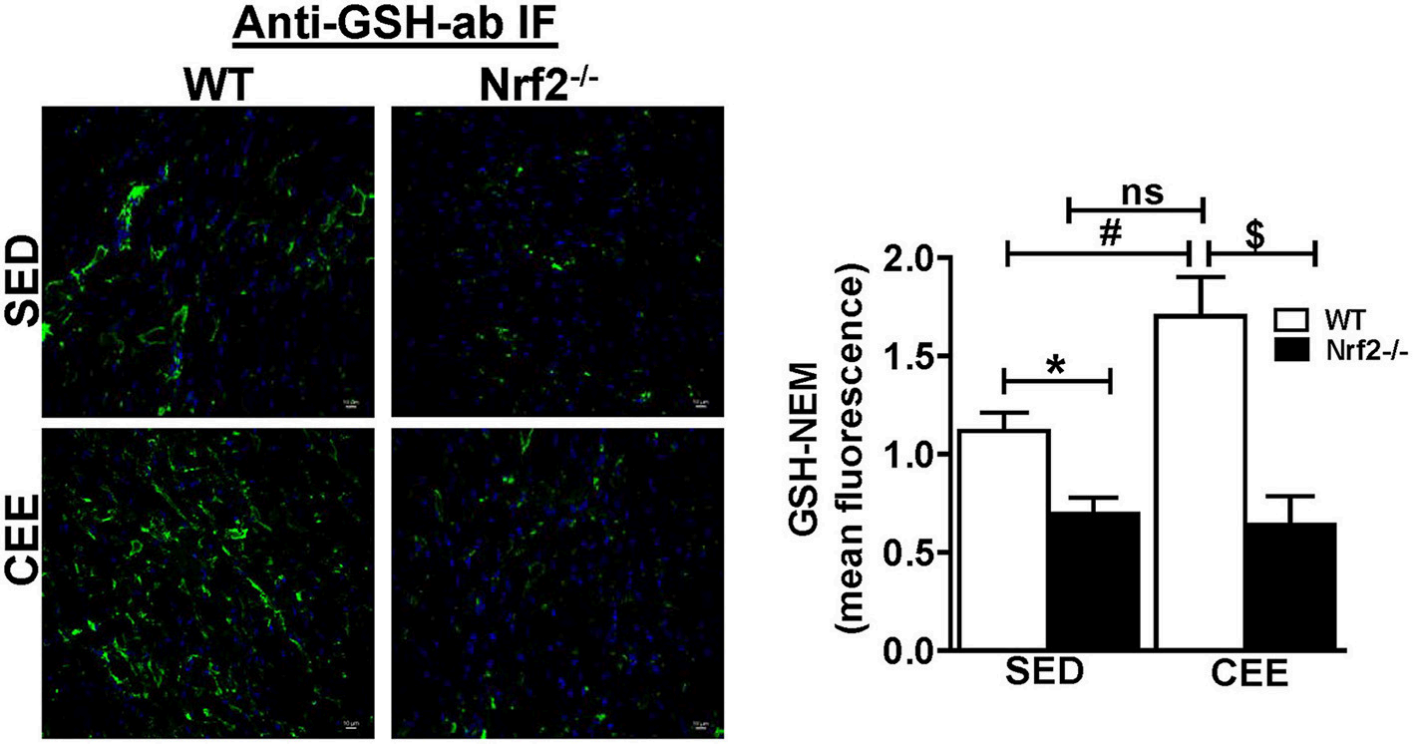
**Prolonged endurance exercise decreases GSH levels in aged Nrf2 deficient mice: WT and Nrf2^**−/−**^mice heart cryosections were fixed and permeabilized for immunofluorescence staining for GSH-NEM**. Permeabilized heart sections were incubated with NEM (10 mM) followed by incubation with GSH-NEM antibodies and processed for imaging with 60X magnification. Captured images were quantified for fluorescence intensity and mean fluorescence intensity was represented as histogram (*n* = 4/group). Experiments were analyzed using one way ANOVA followed by Tukey multiple comparison tests and differences between the means were considered statistically significant if *P* < 0.05 (^*^ vs. WT-SED, ^#^ vs. Nrf2^−/−^-SED, ^*$*^ vs. WT CEE).

Physiological aging is associated with increased ROS and decreased antioxidant signaling and augmentation of GSH homeostasis plays a critical role in preserving redox balance and is shown to exert cardio-protective effect (Tocchetti et al., 2012; Tullio et al., 2013). However, the effect of CEE induced changes in the status of myocardial GSH during physiological (WT) and pathological (loss of Nrf2) is not clearly known. Our immunofluorescence analysis for GSH indicate that under basal conditions, Nrf2^−/−^ hearts showed a significant reduction in the fluorescence signals for GSH by ~40% (Figure 3). Whereas, Nrf2^−/−^ hearts subjected to CEE showed a pronounced decrease in GSH signals by ~65% in relation to the WT mice that was subjected to CEE. However, the GSH green fluorescence signals were increased in old WT mice subjected to CEE indicating the mice undergoing normal aging is able to mount a stress-response by increasing GSH levels in the myocardium that is due to intact Nrf2. These results showed that age associated Nrf2 impairment alters the redox system distinctly in basal and stress conditions based on the level of Nrf2 presence.

### Sustained exercise stress increases ROS mediated protein radical formation in aging Nrf2 deficient mice

**Figure 4:**
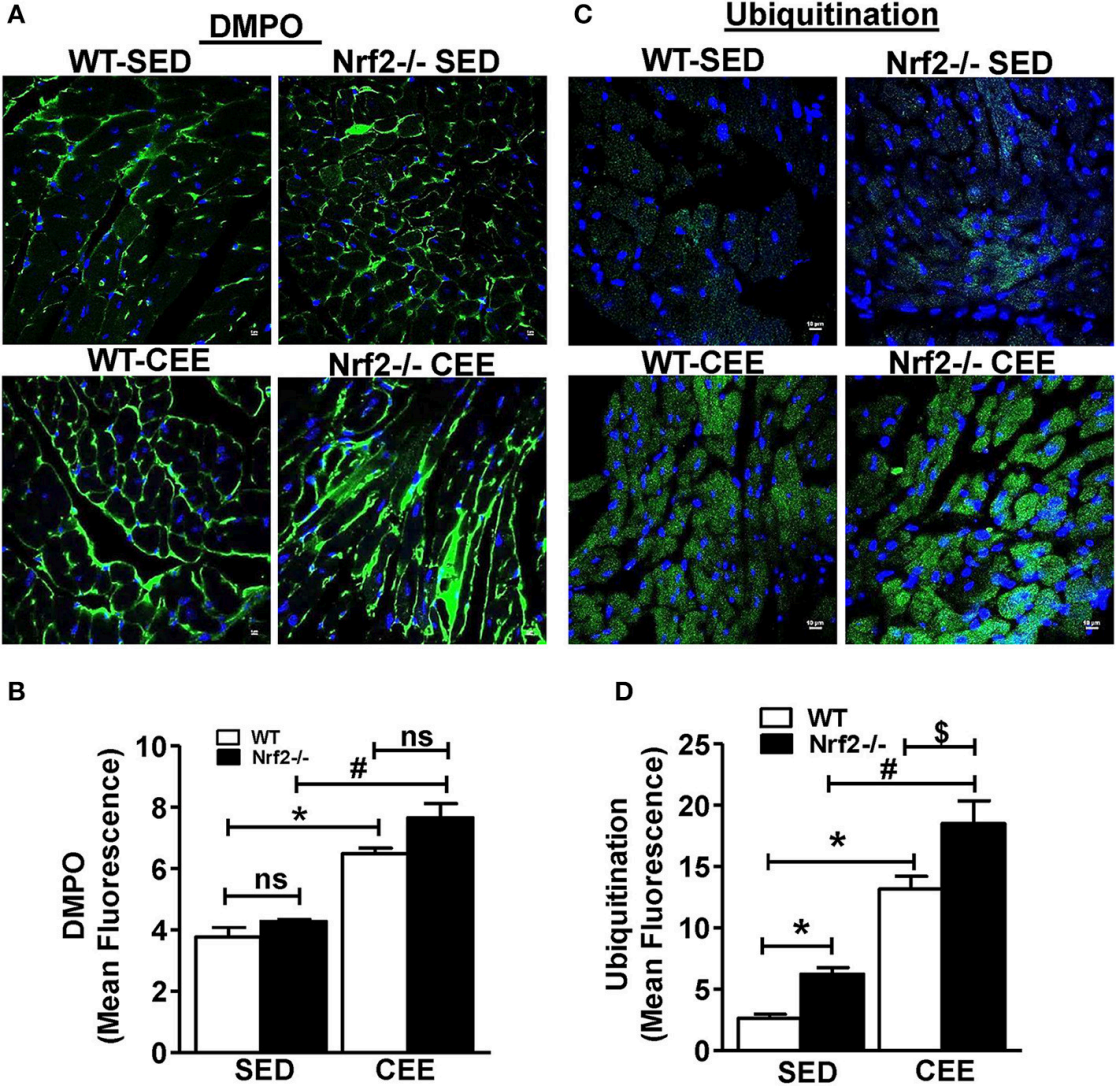
**Chronic endurance exercise induces pronounced oxidative stress and accumulation of ubiquitinated proteins in Nrf2^**−/−**^ mice: DMPO was injected to sedentary and exercised mice 2 h prior to sacrifice**. Cryo-sections from hearts of WT and Nrf2^−/−^ mice were fixed with paraformaldehyde and processed for detecting the **(A,B)** DMPO–protein radical-derived nitrone adducts and **(C,D)** ubiquitin protein conjugates by immunostaining with anti-DMPO and anti-ubiquitin antibodies respectively. The fluorescent images were captured at 60X magnification using confocal microscopy, DAPI staining shows nucleus in blue color. Fluorescence intensity was measured using ImageJ software and the mean fluorescence intensity was represented as mean ± *SD, n* = 4/group. Experiments were analyzed using one way ANOVA followed by Tukey multiple comparison tests and differences between the means were considered statistically significant if *P* < 0.05 (^*^ vs. WT-SED, ^#^ vs. Nrf2^−/−^-SED, ^*$*^ vs. WT CEE).

To investigate whether loss of Nrf2 can lead to protein radical formation in aged mice under basal and stress conditions, we injected the WT and Nrf2^−/−^ mouse with 5,5-Dimethyl-1-pyrroline N-oxide (DMPO), a spin trap that reacts specifically with all type of radicals (O-, N-, S-, and C-centered radicals) followed by staining with anti-DMPO antibodies. A mild to moderate increase in the tissue DMPO reactivity (Green signal) was revealed by immunofluorescence in the hearts from Nrf2^−/−^ when compared with WT mice under basal conditions (Figure 4A). Interestingly, WT and Nrf2^−/−^ mice subjected to chronic EE displayed increased DMPO mean fluorescence intensity by 60 and ~100% respectively in a cross-section of the heart compared to its sedentary counterparts. This result suggests that that chronic EE can profoundly increase the age-associated augmentation of free radical-mediated oxidation in the Nrf2^−/−^ myocardium (Figures 4A,B). Intriguingly, though the GSH levels were increased in old WT mice subjected to CEE (~40%; Figure 3; lane 1 vs. lane 3), the induction level of oxidative modification appears to be higher than the GSH levels (60%; Figure 4B; lane 1 vs. lane 3).

### Chronic endurance exercise stress exacerbates protein ubiquitination in the aged Nrf2^−/−^ mouse myocardium

The activity of ubiquitin-proteasome systems that plays a major role in the degradation of misfolded proteins and the level of oxidative damage together coordinate the events associated with proteostasis (Di Domenico et al., 2014; Díaz-Villanueva et al., 2015; Labbadia and Morimoto, 2015). We next tested whether the oxidative environment that was resulting from of chronic EE in Nrf2^−/−^ mice increase the ubiquitinated proteins using ubiquitin-ab fluorescent staining. Under the sedentary state, increased ubiquitin staining was observed in Nrf2^−/−^ mice relative to WT (Figures 4C–D) indicating a role for Nrf2 in age-associated protein ubiquitination (Miller et al., 2012). In addition, we observed a significantly intense ubiquitin staining in WT mice after chronic EE compared to the sedentary WT and Nrf2^−/−^ mice. Notably, the ubiquitin staining and the quantitative assessment of the sections from Nrf2^−/−^ mice subjected to chronic EE demonstrates a more pronounced increase in ubiquitin immunoreactivity compared to WT littermate subjected to chronic EE. More nuclear ubiquitination was also observed (Figure 4C). Collectively, these observations suggest that abundant changes in protein ubiquitination are associated with the loss of Nrf2 and increased myocardial oxidative injury.

### Absence of Nrf2 in aged mice exacerbates pathological ventricular remodeling in response to chronic endurance exercise

**Figure 5:**
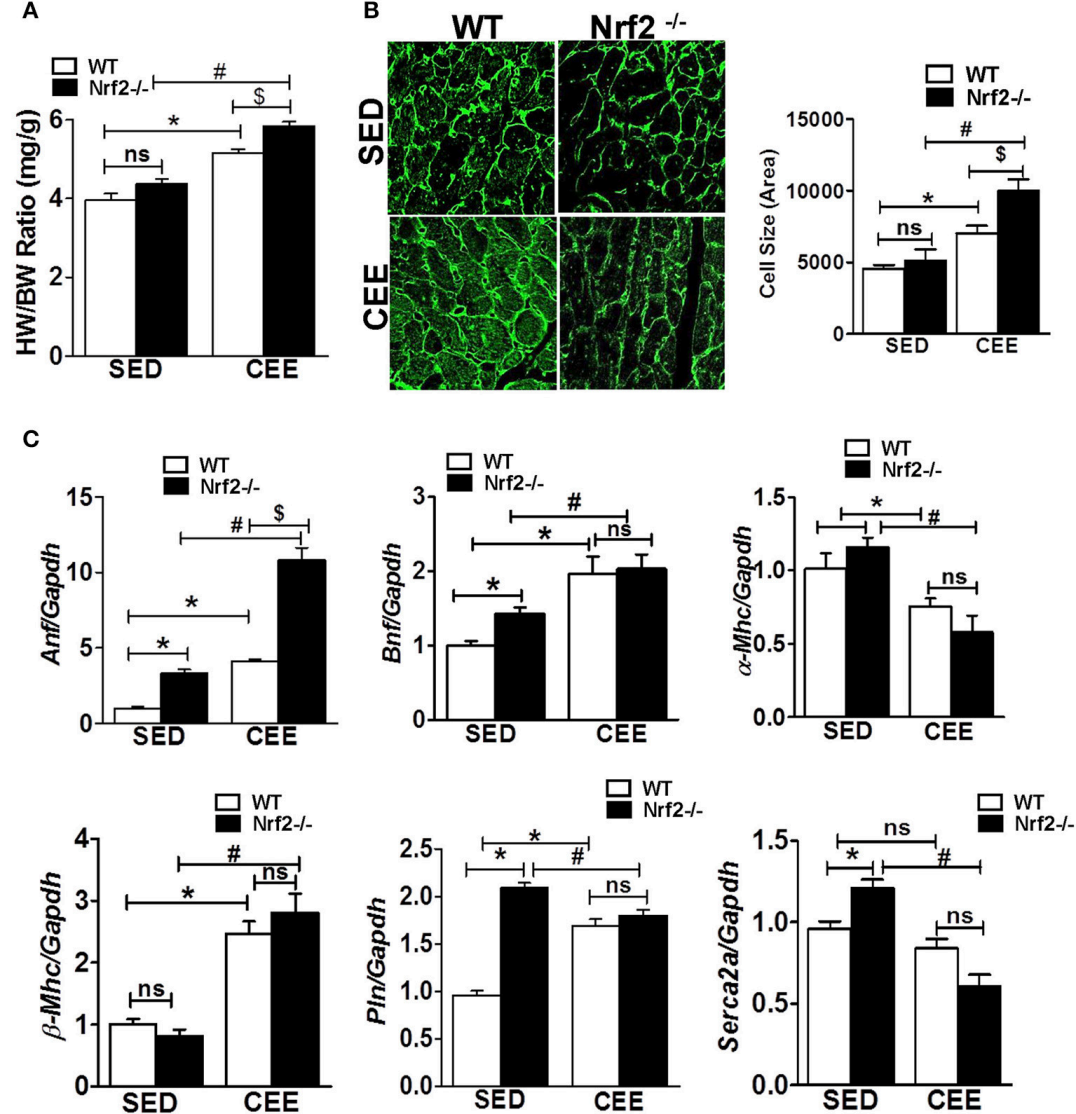
**Cardiac remodeling and myocardial hypertrophy gene expression in WT and Nrf2^**−/−**^ mice subjected to endurance exercise: (A)** Heart weight/ body weight ratio was calculated from the data collected upon sacrifice, **(B)** heart cryosections were stained with wheat germ agglutinin (WGA) and imaged using Nikon eclipse at 20x magnification. Cell size was measured by quantifying the cardiomyocyte area using ImageJ software, (*n* = 35–50 cells/group). **(C)** RT-qPCR was performed for hypertrophic gene markers (*Anf, Bnf*, α*-Mhc*, β*-Mhc, Pln*, and *Serca2a*) in sedentary and exercise mice (WT and Nrf2^−/−^). *n* = 4/group. Histograms show mean ± *SD* and statistical analysis was determined by one way ANOVA followed by Tukey multiple comparison tests (^*^P < 0.05 vs. WT-SED, ^#^*P* < 0.05 vs. Nrf2^−/−^-SED, ^*$*^*P* < 0.05 vs. WT CEE).

Since the loss of Nrf2-antioxidant signaling is closely connected to pathological remodeling of the myocardium, we next assessed whether the chronic EE exaggerate the ventricular remodeling in the aged myocardium of Nrf2 knockout animals. Under the sedentary state, the heart weight/body weight ratio was statistically not different between Nrf2^−/−^ vs. WT mice. However, chronic EE significantly increased the HW/BW ratio in Nrf2^−/−^ mice relative to WT suggestive of cardiac remodeling upon loss of Nrf2 (Figure 5A). Next, we examined the role of Nrf2 in chronic EE-induced alterations in cardiac myocyte size/structure using wheat germ agglutinin immunostaining. Under the sedentary state, we found no structural/size changes in cardiomyocytes between WT and Nrf2^−/−^ mice (Figure 5B). However, a significant increase in cell size was noted in WT subjected to chronic EE which was further increased in Nrf2^−/−^ mice (Figure 5B). We next measured the myocardial expression of hypertrophy gene markers. A significant increase in the expression of hypertrophy markers (*Anf* and *Bnf*) with no change in α*-Mhc* and β*-Mhc* in Nrf2^−/−^ vs WT mice was observed in the physiological sedentary state. However, under chronic EE, all the studied hypertrophy markers except α*-Mhc* were significantly increased in both WT and Nrf2^−/−^ mice (Figure 5C). Interestingly, the expression of α*-Mhc* was significantly downregulated in chronic EE animals when compared to its sedentary counterparts. The calcium regulatory markers, *Pln* and *Serca2a* were considerably altered in Nrf2^−/−^ mice in relation to its sedentary WT mice suggesting the impact of Nrf2 on calcium regulatory system during aging. Following chronic EE, Nrf2^−/−^ mice displayed a significant reduction in the *Serca2a* expression below the baseline, while the *Pln* was slightly decreased when compared to the corresponding sedentary but was still increased when compared to the WT indicating a possible disturbance in calcium handling. Altogether, these observations suggest that both WT and loss of Nrf2 in aged mice can significantly promote the CEE-induced remodeling process, but the loss of Nrf2 exerts a differential and more pronounced effect vs. the WT (HW/BW; cell size, *Anf*).

### Endurance exercise training impairs the electrophysiological changes associated with atrioventricular block in aged Nrf2 knockout mouse

**Figure 6:**
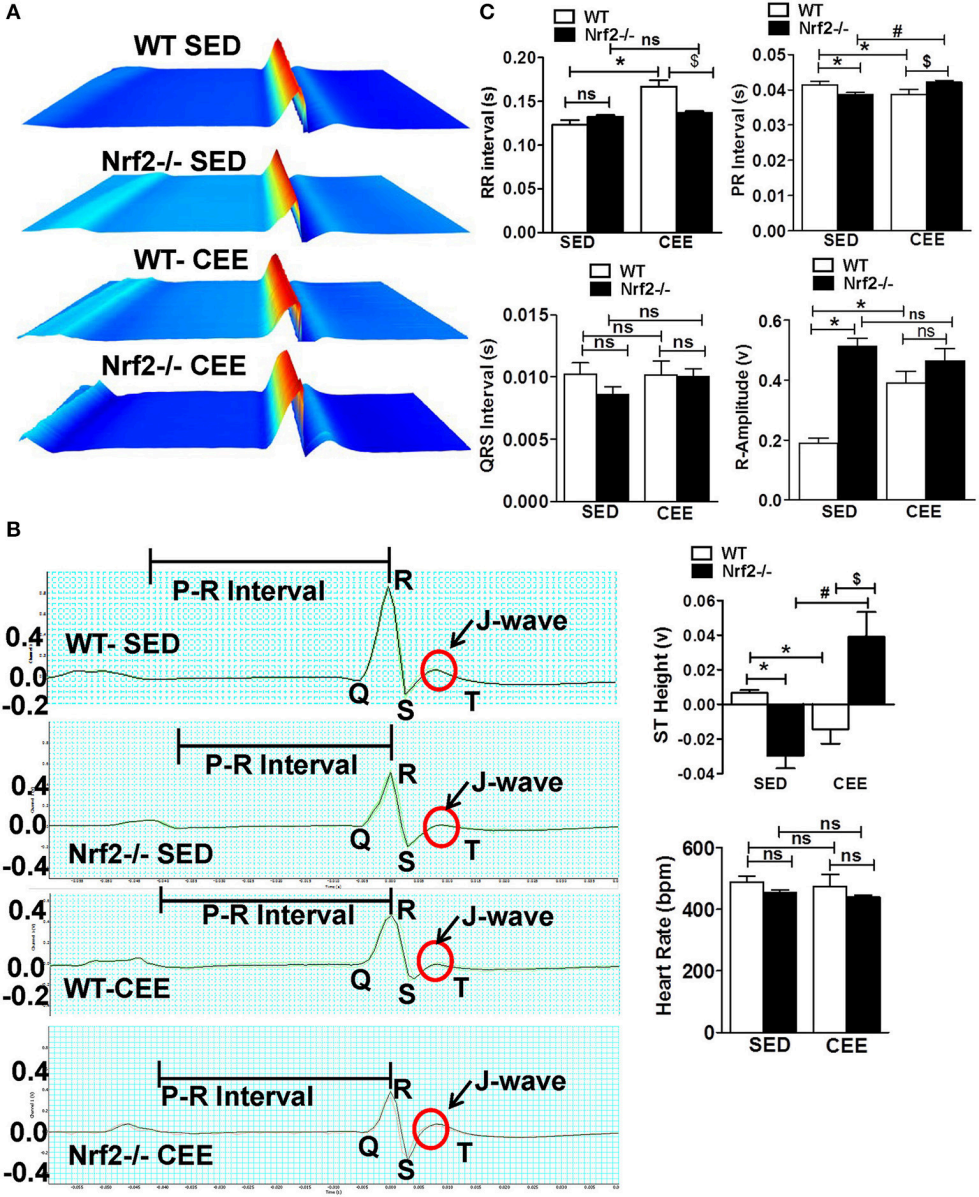
**Electrophysiological changes in response to endurance exercise in WT and Nrf2^**−/−**^ mice:** Electrocardiograms were measured in sedentary and CEE mice for 5-min using iWorx IX/228S data acquisition unit. **(A)** Waterfall plots displaying changes in ECG traces from 0 to 5 min. **(B)** A representative electrocardiogram tracings of WT and Nrf2^−/−^ mice under sedentary and CEE conditions. **(C)** Quantitative evaluation of PR interval, RR interval, R amplitude, QRS interval, ST height, and heart rate were performed using Labchart 8 pro software in aged WT and Nrf2^−/−^ mice in response to prolonged endurance exercise. *n* = 4/group. Histograms show mean ± *SD* and values with different symbols differ statistically significant with each other at *P* < 0.05 as assessed by one way ANOVA followed by Tukey multiple comparison tests (^*^P < 0.05 vs. WT-SED, ^#^*P* < 0.05 vs. Nrf2^−/−^-SED, ^*$*^*P* < 0.05 vs. WT CEE).

We next used surface electrocardiographical (ECG) analysis to study the cardiac function under sedentary and chronic exercise condition. Under the sedentary condition, loss of Nrf2 significantly shortened the PR interval (atrioventricular conduction interval) along with R-wave enlargement and decrease in the ST height in relation to aged WT mice (Figure 6). While the QRS duration and RR-interval (heart rate) was similar between the genotypes at the sedentary state. After 6 weeks of chronic EE, WT mice exhibited a significant change in R-amplitude, PR interval, RR-interval, and ST heights compared to the sedentary mice. Interestingly, chronic EE led to pronounced ST segment elevation and J wave elevation in Nrf2^−/−^ aged mice. Further, RR intervals (which represent the intervals between successive heart beats) were shortened in the Nrf2^−/−^ knockouts that were chronically stressed with CEE in comparison to the WT counterparts. Altogether, these results suggest that chronic EE alters the electrophysiological characteristics of atrioventricular block and ventricular hypertrophy in aging Nrf2^−/−^ animals.

### Echocardiogram reveals altered cardiac function in aged Nrf2^−/−^ mice in response to chronic endurance exercise

**Figure 7:**
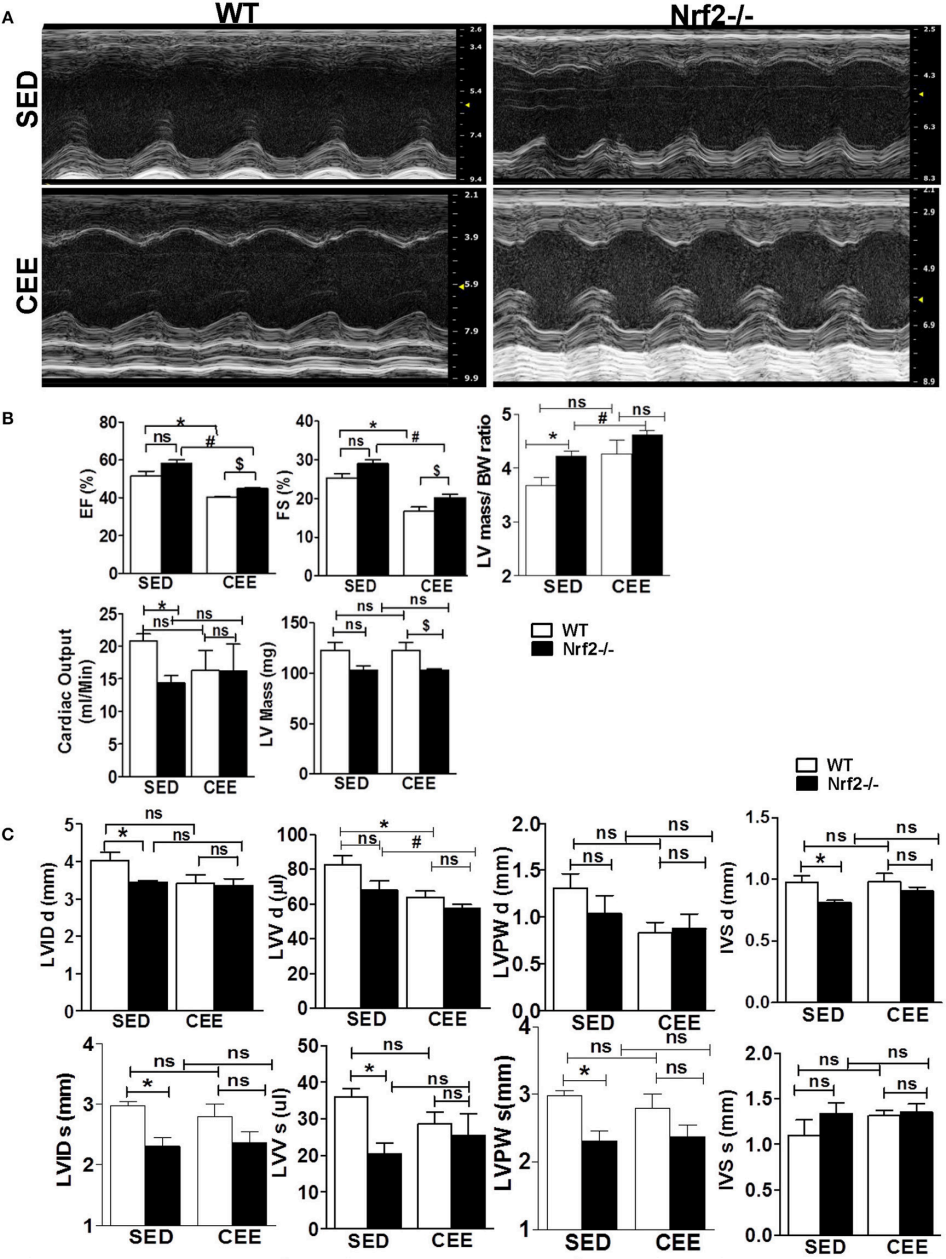
**Echocardiographic changes in Nrf2^**−/−**^ mice after chronic endurance exercise load: Echocardiography was performed using the Vevo2100 System (Visual-Sonics) with high-resolution (38 MHz)**. B-mode and M-Mode images were used to calculate the Echocardiography parameters. **(A)** Representative M-Mode echocardiography images of Nrf2^−/−^ mice and the wild-type littermate control under sedentary and endurance exercise stress. **(B,C)** Measurements of the ejection fraction (EF), fractional shortening (FS), left ventricular volume (LVV), LV weight, cardiac output, Interventricular septal diameter, Left ventricular posterior wall diameter and LV internal diameter (LVID d) are depicted in the histograms as mean ± *SD, n* = 4–6/group. Statistical analyses were performed using one way ANOVA followed by Tukey multiple comparison tests (^*^P < 0.05 vs. WT-SED, ^#^*P* < 0.05 vs. Nrf2^−/−^-SED, ^*$*^*P* < 0.05 vs. WT CEE).

Next, to assess the morphological and functional consequences following chronic EE in the hearts of aged WT and Nrf2^−/−^ mice, we performed M-mode echocardiographic analysis. Representative echocardiographic images are shown in Figure 7A. The echocardiographic indices corresponding to systolic function such as EF and fractional shortening (FS), were not significantly different among the genotypes under sedentary state, whereas measures assessing left ventricular size such as LV weight, left ventricular posterior wall diameter and interventricular septal diameter, left ventricular internal diameter, cardiac output, LV weight/BW ratio, and left ventricular volume (LVV) were significantly altered (Figures 7B,C). Some of these systolic and diastolic parameters (left ventricular posterior wall diameter, left ventricular posterior wall diameter, interventricular septal diameter, left ventricular internal diameter, and cardiac output) were not significantly altered between WT and Nrf2^−/−^ mice subjected to CEE. However, WT mice subjected to chronic EE significantly suppressed the EF, FS, and LVV d volume when compared to its sedentary counterparts. Further, chronic EE significantly increased EF and FS in the Nrf2^−/−^ mice as compared to WT (Figure 7B) indicating that an impairment of systolic function. Notably, the measures of LV size such as LV weight, LVV d volumes were significantly altered in chronically EE stressed Nrf2^−/−^ animals compared to its sedentary Nrf2^−/−^ mice. Overall, these results suggest that the loss of Nrf2 significantly affects the cardiac remodeling in response to CEE stress.

### Chronic endurance exercise stress induces diastolic dysfunction in Nrf2^−/−^ mice

**Figure 8:**
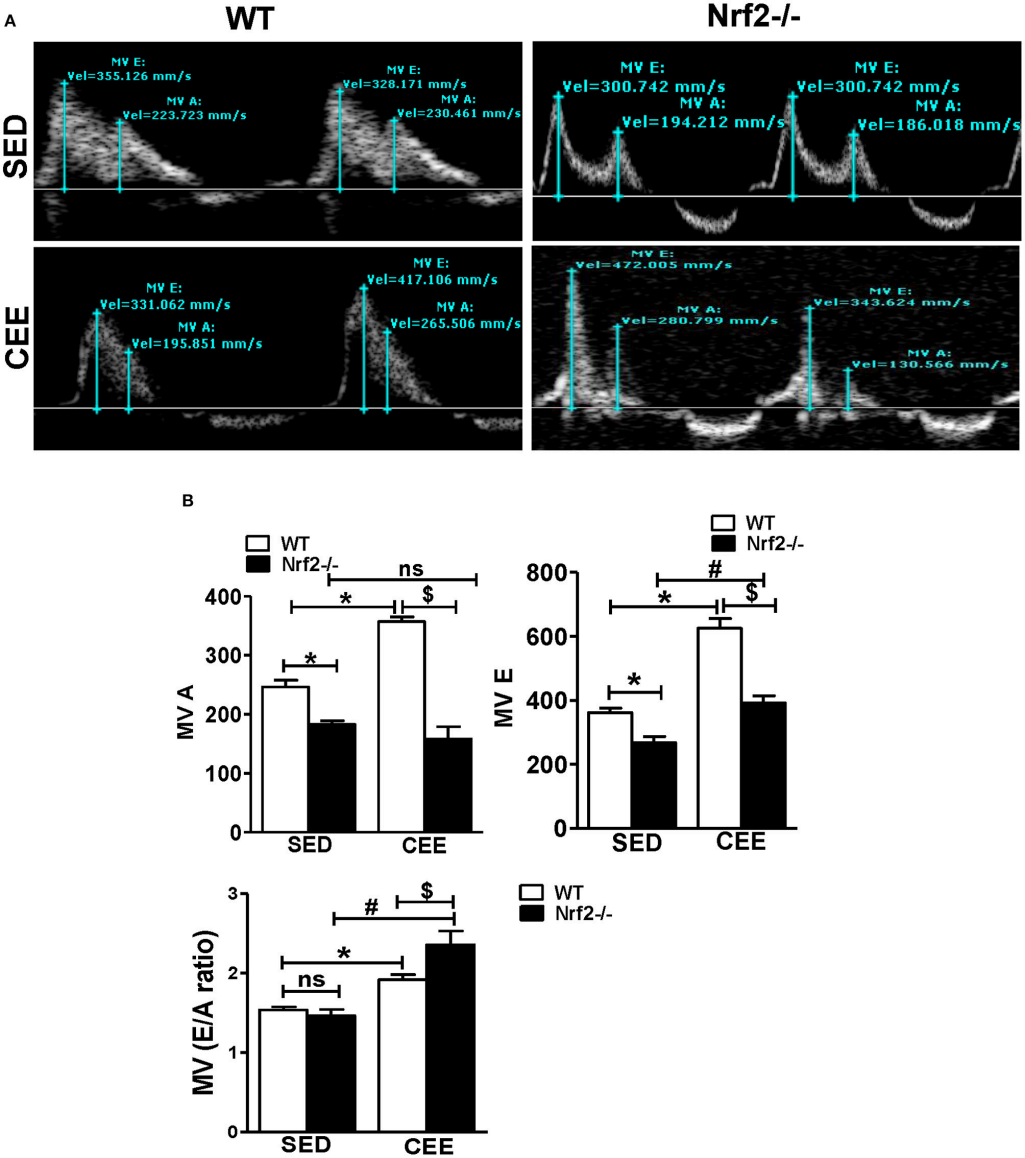
**Chronic endurance exercise prompts diastolic dysfunction in aged mice: Diastolic dysfunction was measured by placing the pulse wave Doppler across the mitral valve and mitral valve inflow was measured in aged WT and Nrf2^**−/−**^ mice under sedentary and exercise condition. (A)** Representative pulse wave Doppler images showing transmitral blood flow velocity patterns, **(B)** Mitral valve E-wave (early filling) and A-wave (late diastolic filling) patterns were measured using the mitral valve images and the mitral inflow pattern was derived from MV E/A ratio. The bars in the histogram represent the mean ± *SD* for each sample and the statistical difference is indicated as *p*-value obtained from the one-way ANOVA followed by Tukey multiple comparison tests (^*^P < 0.05 vs. WT-SED, ^#^*P* < 0.05 vs. Nrf2^−/−^-SED, ^*$*^*P* < 0.05 vs. WT CEE).

Diastole is a crucial phase of the cardiac cycle, requiring proper contraction, and relaxation. While acute exercise is well-documented to produce beneficial adaptations in heart rate and myocardial excitation-contraction coupling, CEE, or prolonged endurance exercise tends to generate pathological increases in cardiac contractility resulting in heart failure (Maron et al., 1996; Piña et al., 2003; O'Keefe et al., 2012; Fabritz et al., 2014). Thus, we sought to determine, if there are any changes in the diastolic function following CEE stress using pulse wave color Doppler analyses of the mitral valve. The representative doppler profile illustrated in Figure 8A and the corresponding quantification of the characteristic waves in Figure 8B indicates that under sedentary state, the Nrf2^−/−^ mice exhibit a significant fall in both the MV E that represents an early transmitral flow (diastolic filling) and MV A values that represent a late flow with atrial contraction. Since both the E and A wave were reduced in Nrf2^−/−^ mice, the relative contribution of each of them that was represented by E/A ratio was not significantly different. Further, there was a significant increase in MV E, MV A values, and MV E/A ratios in WT mice subjected to chronic EE suggesting possible diastolic dysfunction. Interestingly, when the Nrf2^−/−^ aged mice was subjected to endurance exercise stress, the MV E appears to look relatively normal which actually is a pseudo-normalization pattern, a characteristic of disease progression (Appleton et al., 1988) and MV A was found to be significantly reduced resulting in more pronounced increase in E/A when compared with WT mice subjected to chronic EE (Figure 8B). These results suggest that loss of Nrf2 in aged animals when subjected to chronic EE could exhibit restrictive filling pattern (E/A >1.5) along with profound diastolic dysfunction (Appleton et al., 1988; Bella et al., 2002; Nagueh et al., 2009).

## Discussion

Redox homeostasis is critical for the regulation of basal cellular signaling, but an acute or chronic change toward extreme oxidative side together with a compromised antioxidant system is anticipated to be harmful to the myocardium (Neri et al., 2007). In the present study, we investigated whether genetic ablation of the master regulator of antioxidant genes namely, nuclear erythroid derived factor-2 (Nfe2L2 or Nrf2) could prompt pathological structural remodeling of the ventricle in a human equivalence of late middle-aged mice experiencing CEE. Our findings indicate that ablation of Nrf2 leads to (i) diminished levels of a subset of antioxidant genes and proteins in the heart, (ii) augmented OS associated with a profound increase in the extent of ubiquitination observed in Nrf2^−/−^ mice subjected to CEE, (iii) pronounced cardiac remodeling, (iv) abnormal cardiac electrophysiological characteristics following CEE compared to its WT counterparts, (v) both WT and Nrf2^−/−^ mice exhibit significant decrease in systolic function (EF) post CEE, but Nrf2^−/−^ showed pronounced diastolic dysfunction. Altogether, our results suggest that ablation of Nrf2 in mice of advancing age is incapable of achieving the complete benefits to endurance exercise as that of the age-matched WT myocardium.

In sedentary humans, the Nrf2 signaling is impaired in association with augmented OS in skeletal muscle with aging (Safdar et al., 2010). Previous studies have reported that increased ROS/RNS production contribute to several age related cardiovascular diseases in humans (Son, 2012; Wu et al., 2014). Here, >22 months old WT mice displayed increased cardiac hypertrophy and decreased systolic function after CEE, which was further elevated in the Nrf2^−/−^ mice due to the loss of stress-response defense mechanisms coupled with enhanced oxidative burden. Increase in global ubiquitination indicating a possible activation of ubiquitin-proteasome degradation system (UPS) can supply an excess of amino acids and other metabolites resulting in hypertrophy and diastolic dysfunction in the heart of Nrf2^−/−^ mice subjected to chronic CEE (Patil et al., 2012). Therefore, an optimal expression and function of Nrf2 signaling are vital to preserve myocardial antioxidant defense and homeostatic redox at stress conditions.

It is widely believed that diastolic dysfunction occurs independently or preceding a systolic dysfunction (Cazzaniga et al., 2007; Fontes-Carvalho et al., 2015; Manolas, 2015), we herein, found that the Nrf2^−/−^ mice with systolic dysfunction exhibited a higher degree of diastolic dysfunction after CEE in relation to WT mice. Previously, a co-occurrence of systolic and diastolic dysfunction has been noted in pathophysiological process of heart failure (Zile and Brutsaert, 2002; Patten and Hall-Porter, 2009; Maharaj, 2012) supporting that the loss of Nrf2 could be associated with both systolic and diastolic dysfunction and accelerate heart failure. Whilst, there remains a debate about whether the cardiac remodeling associated with athletic training, including resistance exercise is beneficial or maladaptive (Vasconcelos et al., 2016), our results suggests that the hypertrophic changes observed in the case of Nrf2 deficiency could be pathological, conversely the similar aged WT mice that also undergoes hypertrophy with insignificant changes in the diastolic function suggesting an adaptive response (Figure 8).

Typically, during exercise the altered action potential duration, conduction velocity, and contractile velocity results in various electrocardiogram (ECG) changes (Hakki et al., 1984; Kawasaki et al., 2006). Interestingly, our finding reveal a distinct pattern of increased R-wave enlargement and RR interval with ST segment down-sloping and J-wave depression in WT mice subjected to CEE. In contrast, ST height and an apparent J-wave elevation with a decrease in RR-interval in Nrf2^−/−^ mice indicating a delayed local conductance of electrical signals, which are attributed to structural remodeling and dysfunction (systolic and diastolic) of the ventricle. The deeper the S wave, the greater the ST-segment elevation, a relation that is observed in patients with left ventricular hypertrophy (Armstrong et al., 2012) and in an acute transmural ischemia (Kléber, 2000; Deshpande and Birnbaum, 2014). ST elevation due to non-ischemic etiologies was also reported up to 15% in the general population (Jayroe et al., 2009; Deshpande and Birnbaum, 2014). Prominent electrocardiographic J wave (elevated J-wave) is shown to be associated with a risk of ventricular fibrillation (VF) (Lévy and Sbragia, 2011; Ohkubo et al., 2012). An elevated J-wave accompanied by ST-elevation is a typical electrocardiographic feature referred to as an early repolarization (ER) pattern (Littmann, 2010; Lévy and Sbragia, 2011) is noted in the Nrf2^−/−^ mice subjected to CEE indicating that loss of Nrf2 could mimic a condition similar to an early repolarization syndrome (ERS). Further, a shortened RR interval that represents the stretch between successive heartbeats in Nrf2^−/−^ mice subjected to CEE in comparison to WT mice clearly indicates the impairment of ventricular rhythm leading to increased chance for ventricular fibrillation in Nrf2^−/−^ mice after CEE. Previous clinical studies in humans reported that the shortened RR interval increases the susceptibility of atrial (AF) and ventricular fibrillation (VF) than the subjects of less susceptibility for AF and VF (Klein et al., 1979).

Systolic performance of the heart was assessed by EF and fractional shortening (FS) (MacIver et al., 2015). Patients with ventricular hypertrophy or hypertrophic cardiomyopathy showed decreased EF and FS (Yoshikawa et al., 2012). Results from the current study showed that decreased cardiac function (EF and FS) coupled with impaired ventricular end diastolic volume in both WT and Nrf2^−/−^ in response to CEE, suggest that CEE stress impairs cardiac function. Of note, an increase in E wave and decreased A wave (MV E/A ratio) in a transmitral pulse-Wave Doppler (Mottram and Marwick, 2005) is profound in the Nrf2^−/−^ mice subjected to CEE compared to the age-matched WT counterparts. This suggests that loss of Nrf2 induces impairment in ventricular filling (restrictive filling pattern) that represents diastolic dysfunction of the heart during the course of CEE (Mottram and Marwick, 2005).

**Figure 9:**
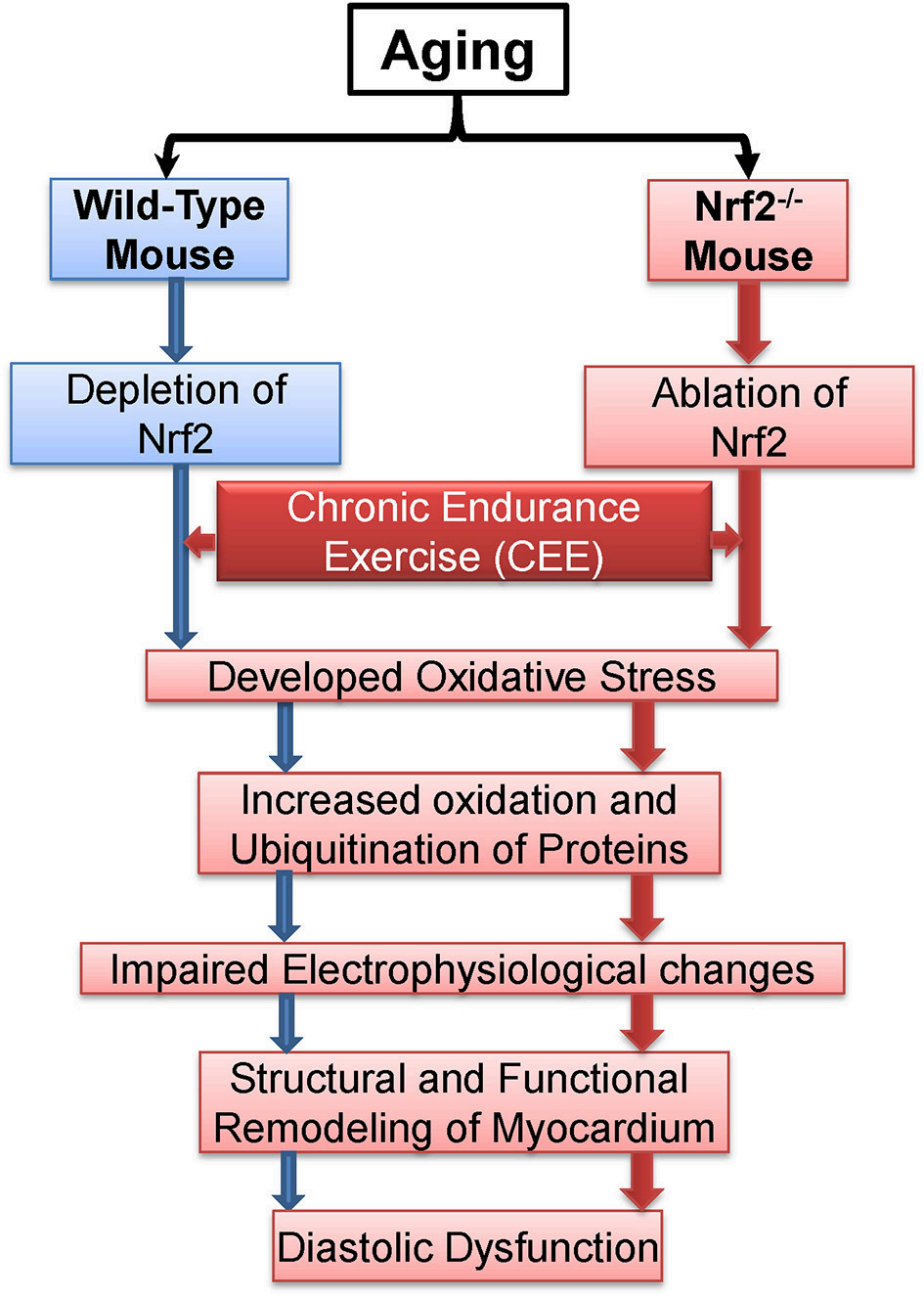
**Proposed model demonstrating the effects of chronic endurance exercise (CEE) on cardiac remodeling under Nrf2 ablation**.

Overall, the central observations presented here suggest that Nrf2 is crucial to avail benefits of exercise (Figure 9). From a mechanistic viewpoint, the loss of Nrf2 signaling associated with aging could have damaging effects besides antioxidant regulation in that it might either directly or indirectly be involved in remodeling of myocardial structure and functional disintegration of the heart in response to repeated stress challenges, e.g., endurance exercise or during loss of adaptive/stress responsive demands (loss of Nrf2) etc. Although both WT and Nrf2^−/−^ mice exhibited comparable myocardial structural remodeling after CEE (hypertrophy), the Nrf2 mice exhibited a pronounced diastolic impairment. This distinction indicates that the combined effect (due to age + Nrf2 ablation) might accelerate heart failure (diastolic dysfunction) in response to chronic stress.

## Author contributions

GS, MN, and RC: Performed the experiments. TS and RS: Interpreted the echocardiography and ECG data. AK and RM: Provided DMPO antibody, designed, and interpreted the DMPO results. JH: Provided directions, edited the manuscript. NSR and MN: Designed the study, interpreted, and wrote the manuscript.

### Conflict of interest statement

The authors declare that the research was conducted in the absence of any commercial or financial relationships that could be construed as a potential conflict of interest.
